# Spatio-Temporal Distribution and Demographic Characteristics of Congenital Heart Defects in Guangdong, China, 2016–2020

**DOI:** 10.3389/fpubh.2022.813916

**Published:** 2022-04-26

**Authors:** Huazhang Miao, Qinghui Zeng, Zengping Shi, Yi Xia, Lushaobo Shi, Dongxue Chen, Pi Guo, Yingxian Zhu, Dong Wang

**Affiliations:** ^1^School of Public Health, Southern Medical University, Guangzhou, China; ^2^School of Health Management, Southern Medical University, Guangzhou, China; ^3^Department of Healthcare, Guangdong Women and Children Hospital, Guangzhou, China; ^4^Department of Preventive Medicine, Shantou University Medical College, Shantou, China

**Keywords:** congenital heart diseases, perinatal infants, spatial, temporal, prevalence

## Abstract

**Background:**

Congenital heart defects are the most common type of birth defects and bring a heavy disease burden in China. Examining the temporal and spatial trends of congenital heart defects epidemics can give some elementary knowledge for succeeding studies.

**Objective:**

To characterize the spatial-temporal patterns of the prevalence of congenital heart defects based on a substantial cohort of the perinatal fetus in south China in 2016–2020.

**Methods:**

This study was a retrospective population-based cohort study conducted in Guangdong, China from 2016 to 2020. Pregnant women and their infants received birth defect surveillance during pregnancy and seven days after delivery in more than 1,900 midwifery hospitals in 21 cities. Perinatal infants with congenital heart defects were identified and enrolled. The prevalence of congenital heart defects was calculated according to cities, years, urban and rural areas, regions of Guangdong, categories of maternal age at delivery, seasons of delivery, and infant's gender.

**Results:**

A total of 8,653,206 perinatal infants and 53,912 total congenital heart defects were monitored in Guangdong, including 46,716 (86.65%) without other defects and 7,736 (13.35%) with other defects. The average prevalence of total congenital heart defects was 62.30/10,000 (95% CI, 61.78/10,000–62.83/10,000), congenital heart defects without other defects was 53.36/10,000 (95% CI, 52.88/10,000–53.85/10,000), and congenital heart defects with other defects was 8.94/10,000 (95%CI, 8.74/10,000–9.14/10,000). From 2016 to 2020, the prevalence of total congenital heart defects was 54.92/10,000, 54.23/10,000, 63.79/10,000, 73.11/10,000, 68.20/10,000, respectively. We observed geographical variations within the prevalence of congenital heart defects. The prevalence of congenital heart defects was much higher in the Pearl River Delta region than in the non-Pearl River Delta region, as well as higher in urban areas than in rural areas.

**Conclusion:**

The findings of this study are helpful to the understanding of the etiology and epidemiology characteristics of congenital heart defects in south China. Our data likely reflect a better estimate of the spatiotemporal trends in congenital heart defects prevalence than reported previously.

## Introduction

Congenital heart defects (CHDs), the most common type of congenital disability, are defined as clinically significant structural heart and/or great vessels disease present at birth (1). About 1/4 of infants born with a heart defect have a critical CHD (2). As medical care and treatment have advanced, infants with CHDs have longer and healthier lives, but the effects of CHDs on infants are lifelong (3). CHDs can develop different health issues in patients over time, depending on their specific heart defect, the number of heart defects they have, and the severity of their heart defect (4).

A previous study showed that the prevalence of CHDs increased worldwide from 1930 to 2009, with the highest prevalence in Asia (93/10,000), followed by Europe (82/10,000), and the lowest in Africa (19/10,000) (5). Further, Wu et al. suggested that the prevalence of CHDs rose in urban and rural populations in Guangdong province from 2008 to 2012 (6). CHDs had become a worldwide major public health problem that seriously affects the quality of the birth population and is particularly severe in Asian regions (5). According to the China Birth Defect Prevention and Control Report, the prevalence of total CHDs in China was 40.95/10,000 in 2011, accounting for 26.70% of all birth defects (7). However, China has a vast territory, and the prevalence of total CHDs shows significant geographical distribution differences between different provinces. Economically developed coastal areas are higher than other inland areas in most cases (8). In addition, previous research on risk factors of four class characteristics or conditions (parental characteristics or conditions, maternal therapeutic drug exposures, parental non-therapeutic drug exposures, and parental environmental exposures.) of a variety of risk factors and that there were significant associations between multiple risk factors and CHDs, but little information is available on the potential adverse effects of these factors on fetal heart rate (4).

Guangdong province, located in southern China, has the largest GDP and resident population among all provinces in China (9). Guangdong began to carry out a birth defects surveillance project in 58 hospitals in 1986, and according to the statistics of 58 hospitals, the prevalence of total CHDs in Guangdong was 52.41/10,000 from 2008 to 2012 (6). Since 2015, Guangdong had initiated a comprehensive prevention and control project for birth defects, the surveillance scope had been extended from the previous 58 hospitals to all midwifery hospitals. All pregnant women and their infants in Guangdong should receive birth defect surveillance during pregnancy and seven days after delivery. When any midwifery hospital found perinatal birth defects, it was necessary to report the “Birth Defect Registration Card” through the Guangdong Provincial Birth Defect Surveillance System (9). Thus, based on the provincial birth defects data from 2016 to 2020, this study can accurately obtain the prevalence of total CHDs and its spatiotemporal and demographic characteristics in Guangdong, which can provide evidence for further prevention and treatment of CHDs or the development of health policies.

## Methods

### Data Sources and Data Collection

We used data from the Guangdong Provincial Birth Defect Surveillance System. We collected the mother's information (age, ethnicity, education level, income level, date of last menstruation, gestational week, gravidity, parity), and the perinatal infant's information (method of delivery, infant gender, birth weight, prognosis status).

The Guangdong Provincial Birth Defect Surveillance System monitored 8,653,206 perinatal infants in Guangdong, China from 2016 to 2020. Among them, we identified and enrolled a total of 53,912 perinatal infants with CHDs (ICD 10: Q20.0–Q26.4), including live births, stillbirths, and neonatal deaths within seven days.

### Statistical Analysis

The prevalence of CHDs was calculated according to years, urban and rural areas, regions of Guangdong, categories of maternal age at delivery, season of delivery, and infant's gender. Descriptive statistics were used to show the essential characteristics of CHDs in infants and their mothers, including the mother's ethnicity, gravidity, parity, income level, education level, and infant's weight, gender, single or multiple births, time of diagnosis, and prognosis status. For differences in the prevalence among characteristics, the χ2 test was used to compare the prevalence of unordered categorical variables. In addition, as for the prevalence trend, because the Cochran-Armitage test is sensitive to population base, compared with linear regression analysis, the Cochran-Armitage test has more advantages when the population is large and the ordered classification variables (years) are minor. Therefore, the Cochran-Armitage test was used to explore the trend in the context of substantial monitoring data of congenital heart defects. A map was used to depict the geographical location of Guangdong in China and the geographical distribution of the four major regions in Guangdong (the Pearl River Delta (PRD) region, Eastern, Western and Northern Guangdong). We used a dot-line diagram to demonstrate the annual prevalence trend of CHDs in Guangdong from 2016 to 2020. We used maps to demonstrate the annual prevalence trend of total CHDs in 21 cities of Guangdong from 2016 to 2020. All statistical analyses were performed within the SPSS v27.0 software, and diagrams and maps were drawn by R v4.1.0 software.

## Results

**Figure 1:**
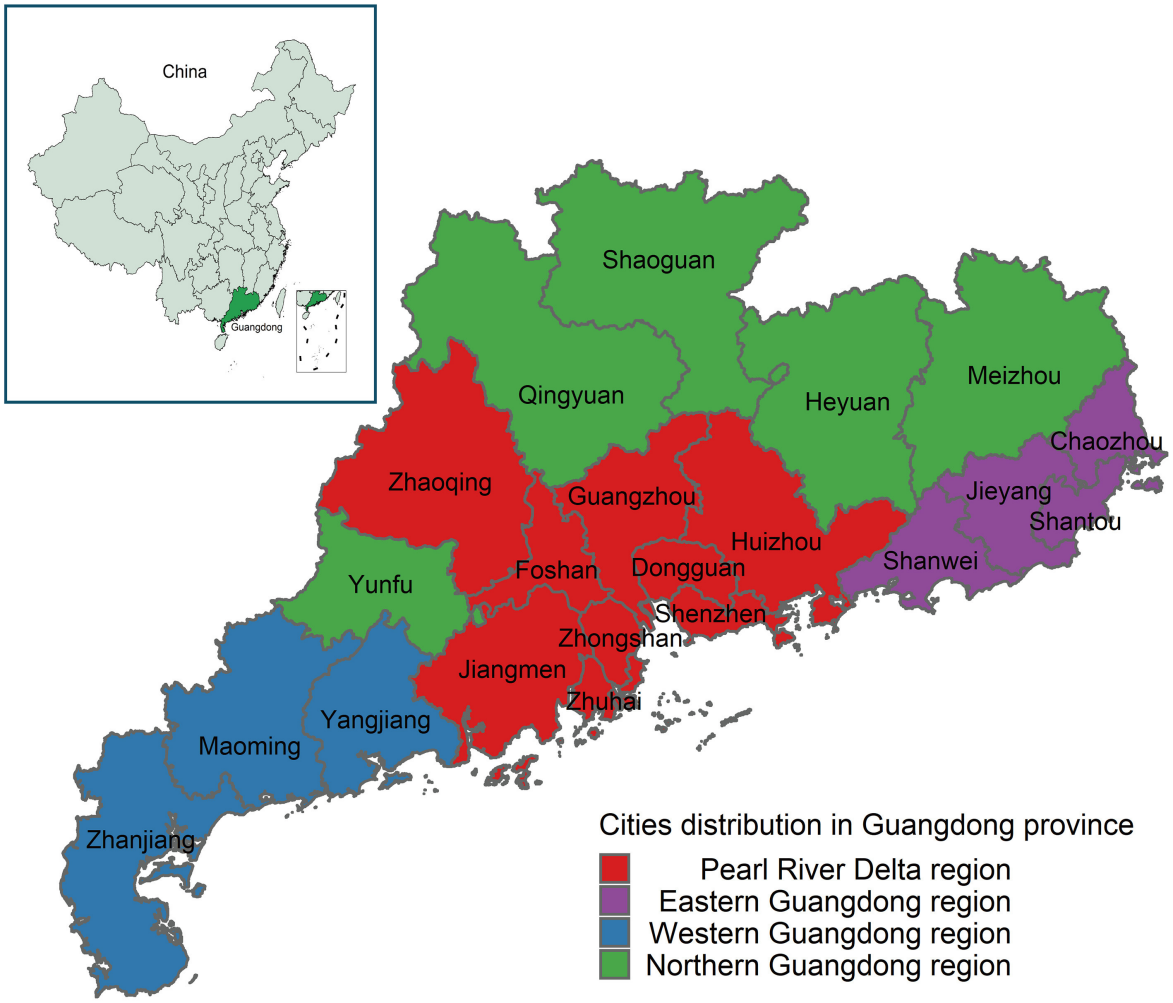
Geographical locations of the study area. Guangdong, which located in southern China, includes 21 cities, and divided into four regions. The Pearl River Delta region includes Guangzhou, Dongguan, Shenzhen, Huizhou, Zhaoqing, Foshan, Zhongshan, Zhuhai, and Jiangmen; Eastern Guangdong includes Shanwei, Jieyang, Shantou, and Chaozhou; Western Guangdong includes Yangjiang, Maoming, and Zhangjiang; Northern Guangdong includes Yunfu, Qingyuan, Shaoguan, Heyuan, and Meizhou.

Figure 1 can manifest the geographical location of Guangdong as well as the four divided regions of Guangdong (Pearl River Delta, Eastern, Western, and Northern). The Pearl River Delta region is a developed region with better medical resources, while Eastern, Western, and Northern Guangdong regions are relatively underdeveloped, with insufficient and uneven distribution of medical resources.

**Figure 2:**
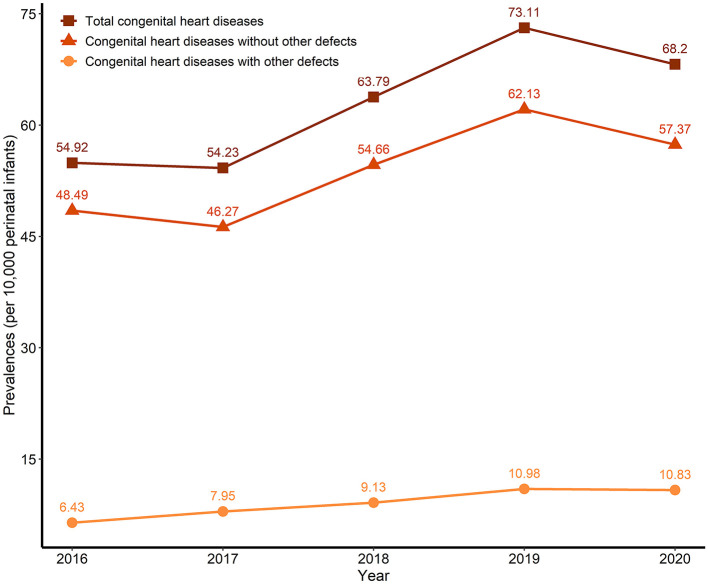
Trends of the prevalence of congenital heart defects (CHDs) (per 10,000 perinatal infants) in Guangdong, China, 2016–2020.

Table 1 and Figure 2 show the prevalence trend of total CHDs in Guangdong from 2016 to 2020. During the study period, a total of 8,653,206 perinatal infants and 53,912 total CHDs were monitored in Guangdong, including 46,716 (86.65%) without other defects and 7,736 (13.35%) with other defects. The prevalence of total CHDs was 62.30/10,000 (95% CI, 61.78/10,000–62.83/10,000). The prevalence of CHDs without other defects was 53.36/10,000 (95% CI, 52.88/10,000–53.85/10,000), and the prevalence of CHDs with other defects was 8.94/10,000 (95%CI, 8.74/10,000–9.14/10,000), the prevalence of CHDs with no other defects was significantly higher than that with other defects (*P* < 0.001). In terms of annual prevalence, the prevalence of total CHDs showed an increasing trend (*P* < 0.001), with the highest prevalence in 2019 (73.11/10,000) and the lowest prevalence in 2017 (54.23/10,000). The trend in the prevalence of CHDs without other defects was similar to that of total CHDs (*P* < 0.001), with the highest prevalence in 2019 (62.13/10,000) and the lowest prevalence in 2017 (46.27/10,000). The prevalence of CHDs with other defects increased year by year (*P* < 0.001), with the highest prevalence in 2019 (10.98/10,000) and the lowest prevalence in 2016 (6.43/10,000).

**Table 1:** Prevalence of congenital heart defects (CHDs) (per 10,000 perinatal infants) in Guangdong, China, 2016–2020.

**Variables^a^**	**Numbers of perinatal infants**	**CHDs without other defects (*****n*** **=** **46,716)**				**CHDs with other defects (*****n*** **=** **7,736)**				**Total CHDs (*****n*** **=** **53,912)**			
		***N^b^***	***Prevalence***	***95%CI^c^***	***P***	***N^b^***	***Prevalence***	***95%CI^c^***	***P***	***N^b^***	***Prevalence***	***95%CI^c^***	***P***
**Years**					<0.001				<0.001				<0.001
2016	1,854,646	8,993	48.49	47.49–49.50		1,193	6.43	6.08–6.81		10,186	54.92	53.86–56.00	
2017	1,932,464	8,942	46.27	45.32–47.24		1,537	7.95	7.56–8.36		10,479	54.23	53.20–55.27	
2018	1,694,639	9,263	54.66	53.56–55.78		1,547	9.13	8.68–9.59		10,810	63.79	62.60–65.00	
2019	1,645,752	10,225	62.13	60.93–63.34		1,807	10.98	10.48–11.49		12,032	73.11	71.81–74.42	
2020	1,525,705	8,753	57.37	56.18–58.58		1,652	10.83	10.32–11.36		10,405	68.20	66.90–69.52	
2016–2020	8,653,206	46,176	53.36	52.88–53.85		7,736	8.94	8.74–9.14		53,912	62.30	61.78–62.83	
**Residential areas**					<0.001				<0.001				<0.001
Urban	4,164,278	37,554	90.18	89.27–91.10		6,186	14.85	14.49–15.23		43,740	105.04	104.06–106.02	
Rural	4,488,928	8,622	19.21	18.81–19.62		1,550	3.45	3.28–3.63		10,172	22.66	22.22–23.10	
**Region of Guangdong** ^d^					<0.001				<0.001				<0.001
PRD	4,997,047	40,253	80.55	79.77–81.34		6,673	13.35	13.04–13.68		46,926	93.91	93.06–94.76	
Eastern GD	1,215,344	939	7.73	7.24–8.23		261	2.15	1.90–2.42		1,200	9.87	9.33–10.44	
Western GD	1,321,727	2,041	15.44	14.78–16.12		275	2.08	1.85–2.34		2,316	17.52	16.82–18.25	
Northern GD	1,119,088	2,943	5.59	5.68–6.11		527	1.05	0.97–1.15		3,470	6.94	6.72–7.18	
**Maternal age at delivery**					<0.001				<0.001				<0.001
<20	236,497	745	31.50	29.30–33.83	<0.001^e^	123	5.20	4.34–6.18	<0.001^e^	868	36.70	34.32–39.21	<0.001^e^
20–24	1,628,600	6,429	39.48	38.52–40.45	<0.001^e^	1,000	6.14	5.77–6.53	<0.001^e^	7,429	45.62	44.59–46.66	<0.001^e^
25–29	3,316,539	16,748	50.50	49.94–51.27		2,680	8.08	7.78–8.39		19,428	58.58	57.76–59.41	
30–34	2,321,374	13,987	60.25	59.26–61.26	<0.001^e^	2,228	9.60	9.21–10.00	<0.001^e^	16,215	69.85	68.78–70.93	<0.001^e^
≥35	1,150,196	8,120	70.60	69.07–72.14	<0.001^e^	1,682	14.62	13.94–15.34	<0.001^e^	9,802	85.22	83.55–86.92	<0.001^e^
**Season of delivery** ^f^					<0.001				<0.001				<0.001
Spring	1,988,879	11,530	57.97	56.92–59.04		2,071	10.41	9.97–10.87		13,601	68.39	67.24–69.54	
Summer	2,139,333	11,687	54.63	53.65–55.63	<0.001^g^	2,111	9.87	9.45–10.30	0.101^g^	13,798	64.50	63.43–65.60	<0.001^g^
Autumn	2,387,406	11,462	48.01	47.14–48.90	<0.001^g^	1,856	7.77	7.43–8.13	<0.001^g^	13,318	55.78	54.84–56.74	<0.001^g^
Winter	2,137,588	11,497	53.78	52.81–54.77	<0.001^g^	1,698	7.94	7.57–8.33	<0.001^g^	13,195	61.73	60.68–62.79	<0.001^g^
**Infants' gender** ^H^					<0.001				<0.001				<0.001
Male	4,598,644	24,343	52.94	52.57–53.60		4,205	9.14	8.87–9.42		28,548	62.08	61.36–62.80	
Female	4,053,112	21,245	52.42	51.72~53.12		3,043	7.51	7.24–7.78		24,288	59.92	59.17–60.68	

a *Chi-square tests were used to compare prevalence for all of these variables and obtained the P-value. Besides, the maternal age and season of delivery of each category were compared with the reference category to obtain the adjusted P-values, which were estimated by the Benjamini & Hochberg method*.

b *N, the number of congenital heart disease*.

c * CI, confidence interval*.

d *Four regions, the Pearl River Delta (PRD) region, Eastern, Western and Northern Guangdong (GD) regions are considered*.

e *Adjusted P-values were obtained by comparing to the reference category (mothers aged 25 to 29 years)*.

f *Spring includes March, April, and May. Summer includes June, July, and August. Autumn includes September, October, and November. Winter includes December, January, and February*.

g *Adjusted P-values were obtained by comparing to the reference category (spring)*.

h *Subjects with missing values and hermaphroditism were excluded from this analysis*.

Table 1 and Supplementary Figure 1 also show the prevalence of CHDs stratified by residential areas, regions of Guangdong, maternal age at delivery, season of delivery, infants' gender. The prevalence of total CHDs was higher in urban residents than in rural residents (105.04/10,000 vs. 22.66/10,000, respectively; *P* < 0.001). The prevalence of total CHDs was highest in the Pearl River Delta region, followed by western Guangdong, eastern Guangdong, and lowest in northern Guangdong (93.91/10,000 vs. 17.52/10,000 vs. 9.87/10,000 vs. 6.94/10,000, respectively; *P* < 0.001). The prevalence of total CHDs increased with maternal age, with the highest in the ≥35 years, followed by the 30–34 years, the 25–29 years, the 20–24 years, and the lowest in the <20 years (85.22/10,000 vs. 69.85/10,000 vs. 58.58/10,000 vs. 45.62/10,000 vs. 36.70/10,000, respectively; *P* < 0.001). The prevalence of total CHDs was highest in spring, followed by summer, winter, and lowest in autumn (68.39/10,000 vs. 64.50/10,000 vs. 61.73/10,000 vs. 55.78/10,000, respectively; *P* < 0.001). Females had a higher prevalence of total CHDs than males (62.08/10,000 vs. 59.92/10,000, respectively; *P* < 0.001). Comparisons between different stratifications of the prevalence of CHDs without or with other defects were similar to that of total CHDs.

Table 2 shows the percentage of CHDs across different stratifications separated by characteristics of mothers and infants. Among the mothers, Han had the highest percentage in terms of ethnicity, followed by ethnic minorities (96.79 vs. 2.44%, respectively; *P* < 0.001). Regarding the number of pregnancies, the percentage of pregnancies ≥2 was the highest, followed by pregnancy one times (67.38 vs. 32.55%, respectively; *P* < 0.001). In terms of parity, the percentage of primiparous women was the highest, followed by multiparous women (55.52 vs. 42.92%, respectively; *P* < 0.001). In terms of season, there was little difference in the four seasons, with the highest percentage in summer, followed by spring and autumn, and the lowest in winter (25.59 vs. 25.23 vs. 24.70 vs. 24.48%, respectively; *P* < 0.001). In terms of income level, the percentage of ≥8,000 yuan was the highest, followed by 4,000–8,000 yuan, and the lowest was <4,000 yuan (53.57 vs. 20.73 vs. 7.76%, respectively; *P* < 0.001). In terms of education level, the percentage of undergraduate and above was the highest, followed by high school, and the lowest was middle school and below (32.82 vs. 26.62 vs. 23.60%, respectively; *P* < 0.001). Among infants with CHDs, in terms of gestational age, the percentage was highest at ≥37 weeks, followed by 28–36 weeks, and the lowest was <28 weeks (63.05 vs. 19.80 vs. 17.04%, respectively; *P* < 0.001). In terms of birth weight, the percentage was highest at 2,500–4,000 g, followed by <2,500 g, and the lowest was ≥4,000 g (56.90 vs. 33.08 vs. 3.59%, respectively; *P* < 0.001). In terms of infant gender, the percentage was highest in males, followed by females, and the lowest was hermaphroditism (52.95 vs. 45.05 vs. 0.17%, respectively; *P* < 0.001). In terms of multiple births, the percentage was higher in singletons than in multiple births (93.69 vs. 5.66, respectively; *P* < 0.001). In terms of time of diagnosis, the percentage diagnosed within seven days after delivery was higher than that diagnosed during pregnancy (69.98 vs. 26.35%, respectively; *P* < 0.001). In terms of prognostic outcome, the percentage of live births was highest, followed by stillbirths, and the lowest was death within seven days after delivery (77.75 vs. 18.03 vs. 0.29%, respectively; *P* < 0.001). Comparisons between different stratifications of the percentage of CHDs without or with other defects were similar to that of total CHDs.

**Table 2:** Percentage of congenital heart defects (CHDs) across different stratifications separated by characteristics of mothers and infants.

**Characteristics**	**CHDs without other defects (*****n*** **=** **46,716)**		**CHDs with other defects (*****n*** **=** **7,736)**		**Total CHDs (*****n*** **=** **53,912)**		***x*** **^2^**	***p***
	***N^a^***	***Percentage***	***N^a^***	***Percentage***	***N^a^***	***Percentage***		
**Maternal ethnicity**							12.81	0.002
Han^b^	44,732	96.88	7,454	96.31	52,186	96.79		
Minorities	1,115	2.41	198	2.60	1,313	2.44		
Un-know	329	0.71	84	1.09	413	0.77		
**Number of pregnancies**							56.04	<0.001
1	15,093	33.16	2,186	28.83	17,279	32.55		
≥2	30,386	66.77	5,389	71.08	35,775	67.38		
Un-know	30	0.07	7	0.09	37	0.07		
**Number of deliveries**							85.44	<0.001
1	25,317	54.83	4,616	59.67	29,933	55.52		
≥2	20,174	43.69	2,963	38.30	23,137	42.92		
Un-know	685	1.48	157	2.03	842	1.56		
**Season of delivery^c^**							44.05	<0.001
Spring	11,530	24.97	2,071	26.77	13,601	25.23		
Summer	11,687	25.31	2,111	27.29	13,798	25.59		
Autumn	11,462	24.82	1,856	23.99	13,318	24.70		
Winter	11,497	24.90	1,698	21.95	13,195	24.48		
**Income level**							58.16	<0.001
<4,000 yuan	3,510	7.60	672	8.69	4,182	7.76		
4,000–8,000 yuan	9,723	21.06	1,454	18.80	11,177	20.73		
≥8,000 yuan	24,844	53.80	4,038	52.20	28,882	53.57		
Un-know	8,099	17.54	1,572	20.32	9,671	17.94		
**Education level**							226.69	<0.001
Middle school or less	10,614	22.99	2,109	27.26	12,723	23.60		
High school	12,254	26.54	2,097	27.11	14,351	26.62		
College or above	15,040	32.57	2,653	34.29	17,693	32.82		
Un-know	8,268	17.91	877	11.34	9,145	16.96		
**Gestational age, weeks**							2292.24	<0.001
<28	6,486	14.05	2,703	34.94	9,189	17.04		
28–36	8,981	19.45	1,696	21.92	10,677	19.80		
≥37	30,661	66.40	3,328	43.02	33,989	63.05		
Un-know	48	0.10	9	0.12	57	0.11		
**Birth weight, grams**							1439.6	<0.001
<2,500	13,864	30.02	3,968	51.29	17,832	33.08		
2,500–4,000	27,609	59.79	3,069	39.67	30,678	56.90		
≥4,000	1,774	3.84	160	2.07	1,934	3.59		
Un-know	2,929	6.34	539	6.97	3,468	6.43		
**Infants' gender**							911.54	<0.001
Male	24,343	52.72	4,205	54.36	28,548	52.95		
Female	21,245	46.01	3,043	39.34	24,288	45.05		
Hermaphroditism	52	0.11	37	0.48	89	0.17		
Un-know	536	1.16	451	5.83	987	1.83		
**Multiple births**							19.61	<0.001
Yes	2,581	5.59	469	6.06	3,050	5.66		
No	43,321	93.82	7,190	92.94	50,511	93.69		
Un-know	274	0.59	77	1.00	351	0.65		
**Time of diagnosis**							2258.84	<0.001
During pregnancy	10,495	22.73	3,710	47.96	14,205	26.35		
Within 7 days after delivery	33,763	73.12	3,966	51.27	37,729	69.98		
Un-know	1,918	4.15	60	0.78	1,978	3.67		
**Prognosis status**							2248.99	<0.001
Live birth	37,500	81.21	4,418	57.11	41,918	77.75		
Stillbirth	7,104	15.38	2,615	33.80	9,719	18.03		
Dead within 7 days after delivery	106	0.23	48	0.62	154	0.29		
Un-know	1,466	3.17	655	8.47	2,121	3.93		

a *N, the number of congenital heart disease*.

b *Han nationality is the most populous ethnic group in China*.

c *Spring includes March, April, and May. Summer includes June, July, and August. Autumn includes September, October, and November. Winter includes December, January, and February*.

**Figure 3:**
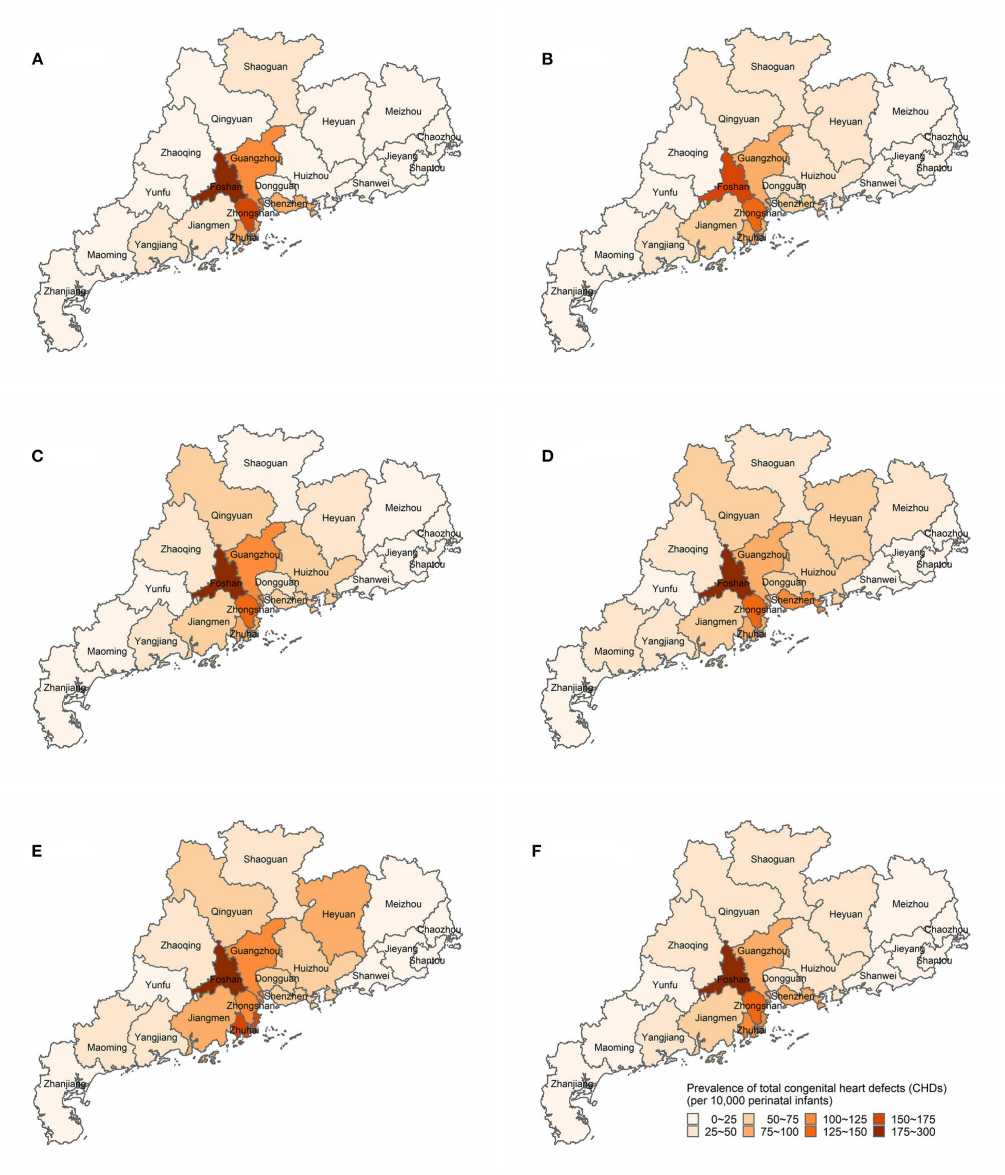
Spatial distributions of the prevalence of total congenital heart defects (CHDs) (per 10,000 perinatal infants) in Guangdong, China, 2016–2020. **(A)** Spatial distributions of total CHDs prevalence in 2016. **(B)** Spatial distributions of total CHDs prevalence in 2017. **(C)** Spatial distributions of total CHDs prevalence in 2018. **(D)** Spatial distributions of total CHDs prevalence in 2019. **(E)** Spatial distributions of total CHDs prevalence in 2020. **(F)** Spatial distributions of total CHDs prevalence in 2016–2020.

Figure 3, Supplementary Figure 2 and Supplementary Table 1 show the spatial distribution of the prevalence of total CHDs in 21 cities of Guangdong from 2016 to 2020. The prevalence of total CHDs among 21 cities in Guangdong showed significant temporal variation and spatial distribution heterogeneity throughout the study period. From 2016 to 2020, the overall prevalence of cities in the Pearl River Delta region was higher than that in other regions. Five cities were higher than the provincial average level (62.30/10,000), which were Foshan (220.05/10,000), Zhongshan (137.28/10,000), Zhuhai (101.59/10,000), Guangzhou (99.74/10,000) and Shenzhen (77.75/10,000), while the other 16 cities were lower than the provincial average level, and the lowest was Chaozhou (3.36/10,000). In 2016, the prevalence of five cities was higher than the provincial average level (54.92/10,000), which were Foshan, Zhongshan, Guangzhou, Zhuhai, and Shenzhen (186.48/10,000, 162.81/10,000, 120.94/10,000, 98.27/10,000, and 88.73/10,000, respectively), and the lowest was Chaozhou (1.82/10,000). In 2017, the prevalence of Foshan, Zhongshan, Guangzhou, Zhuhai, and Shenzhen (168.12/10,000, 126.14/10,000, 91.70/10,000, 83.08/10,000, and 71.62/10,000, respectively) were higher than the provincial average level (54.23%), and the lowest was Chaozhou (1.63/10,000). In 2018, the prevalence of Foshan, Zhongshan, Guangzhou, Zhuhai, and Shenzhen (246.53/10,000, 140.52/10,000, 100.05/10,000, 89.15/10,000, and 64.04/10,000, respectively) were higher than the provincial average level (63.79/10,000), and the lowest was Shanwei (2.78/10,000). In 2019, the prevalence of Foshan, Zhongshan, Shenzhen, Zhuhai, and Guangzhou (280.46/10,000, 140.38/10,000, 106.43/10,000, 96.83/10,000, and 82.50/10,000, respectively) were higher than the provincial average level (73.11/10,000), and the lowest was Shanwei (1.93/10,000). In 2020, the prevalence of Foshan, Zhuhai, Zhongshan, Guangzhou, Jiangmen, Heyuan, Qingyuan, and Huizhou (232.61/10,000, 161.99/10,000, 112.50/10,000, 105.81/10,000, 97.26/10,000, 83.21/10,000, 71.83/10,000, and 68.66/10,000, respectively) were higher than the provincial average level (68.20/10,000), another 13 cities were lower than the provincial average level, and the lowest was Shanwei (2.95/10,000).

## Discussion

Our study performed a comprehensive analysis of spatial-temporal and demographic characteristics of CHDs in Guangdong from 2016 to 2020. The main finding is that there was heterogeneity in the temporal and spatial distribution of the prevalence of CHDs, and the average prevalence of total CHDs was 62.30/10,000 in Guangdong in the past 5 years. From 2016 to 2020, the prevalence of total CHDs was increasing year by year as a whole, peaking in 2019 and slightly decreasing in 2020, but it was still at a high level. The prevalence of CHDs in urban residents was significantly higher than that in rural residents. There were also significant differences among regions in Guangdong, with the highest prevalence of CHDs in the Pearl River Delta region, followed by the western, eastern, and northern regions of Guangdong. The prevalence of CHDs increased with maternal age at delivery, and the prevalence of ≥35 years was almost twice as higher than the <25 years. The prevalence of CHDs was highest in spring, followed by summer, winter, and lowest in autumn. The prevalence of CHDs was higher in male than female infants.

The comprehensive prevention and control project of birth defects is a public health project that requires a tremendous amount of workforce, material, and financial resources. Guangdong was one of the few provinces in China to carry out this project throughout the province. In our study, the prevalence of total CHDs in Guangdong in 2016–2020 was higher than the prevalence obtained by Wu et al. using the data analysis of 58 hospitals in Guangdong in 2008–2012 (62.30/10,000 vs. 52.41/10,000, respectively), and was more accurate and comprehensive (6). Previous studies have shown that the global prevalence of CHDs is generally estimated to be 80/10,000 worldwide, but there was wide variation between countries or regions (5, 10). A study based on the European Surveillance of Congenital Anomalies Central Database found that the average prevalence of perinatal CHDs in Europe from 2000 to 2005 was about 80/10,000 (11). A Perinatal Health Surveillance Report for Congenital Defects issued by the Public Health Agency of Canada showed that the prevalence of CHDs in Canada was 85/10,000 in 2009 (12). According to the Chinese national report on birth defects (2012), the prevalence of total CHDs in China was 40.95/10,000 (7). The prevalence of total CHDs in Guangdong was slightly lower than that in Europe and the North American regions but slightly higher than the average level in China.

In terms of temporal changes, our study showed that the prevalence of total CHDs in Guangdong slightly decreased in 2017, but began to increase significantly in 2018, reached a peak in 2019, and began to decrease slightly again in 2020. This phenomenon may be related to the universal two-child policy issued by China since 2016 (13). From 2016, the number of perinatal infants began to increase considerably, reaching the peak in 2017, but the policy had not produced a cumulative effect, and the number of perinatal infants decreased year by year from 2018. Due to plenty of perinatal infants, the prevalence of CHDs slightly decreased in 2017. However, with the decrease in the number of perinatal infants and the increase in the elderly pregnant women, the prevalence of CHDs began to increase from 2018.

In terms of spatial distribution, there was significant spatial heterogeneity in the prevalence of total CHDs in Guangdong. Previous studies had shown that the distribution of the disease was not balanced, and there was spatial variability worldwide (5, 10). For instance, the prevalence of total CHDs was usually lower in North Africa, Australia, and some provinces of northern China than in South African states, Central Asia and Southeast Asia. Similarly, there were differences in the spatial distribution of prevalence in 34 provinces in China (8). Compared with some provinces in China, the prevalence of CHDs in Guangdong was lower than that in Shanghai from 2014 to 2015 (187.00/10,000) (14), but higher than that in Hunan province from 2016 to 2019 (46.00/10,000) (15). A meta-analysis study showed that the prevalence of total CHDs in China from 2015 to 2019 was 49.05/10,000, slightly lower than that in Guangdong, which may be related to the phenomenon that China has a vast territory, the prevalence in economically developed areas of the south China was higher than that in the north China, and that in the east China was higher than that in the west China (8). Moreover, the prevalence in the Pearl River Delta region was higher than that in the non-Pearl River Delta region, and the prevalence of CHDs in the areas around the Pearl River Delta region showed dynamic changes, which may be related to the local healthcare level and referral system (16). However, it is worth noting that the prevalence of CHDs in Foshan had remained high, and the prevalence was two or three times the provincial average level. The high prevalence should arouse the vigilance of health administrative authorities, find the relevant influencing factors, and take complementary strategies to reduce the prevalence. The prevalence in Chaozhou and Shanwei in eastern Guangdong were too low, suggesting that it may be that the local surveillance ability was insufficient and the diagnosis and treatment ability of CHDs needs to be further improved.

There were significant differences between maternal age at delivery, urban and rural areas, and infant gender in Guangdong for different stratifications. Our findings indicated that with increasing maternal age at delivery, the prevalence of CHDs increases, which was consistent with a study in the United States as well as the study in Hunan province (15, 17). At the same time, significant risk factors for CHDs in perinatal infants may also include increased parental age, but this speculation does not have sufficient evidence of association (18, 19). In addition, in our study, the prevalence of CHDs was significantly lower in rural areas than in urban areas, and female infants had a lower prevalence of CHDs than male infants, which are consistent with the study in Hunan province (15). This may be due to the insufficient surveillance ability of CHDs in rural areas, or due to the developed transportation network, referral network and tiered diagnosis and treatment system in Guangdong, pregnant women in rural areas mostly choose to visit large hospitals in urban areas when they found fetuses with CHDs (20, 21).

## Strengths and Limitations

The advantage of this study was that based on the data of all midwifery hospitals throughout Guangdong, including live births, stillbirths, and perinatal infants who died within seven days after delivery, the trend and spatial distribution of the prevalence of CHDs over the years can be accurately obtained, which can help provide evidence to formulate targeted public health policies, carry out unique prevention and control projects for CHDs, and improve the prevention awareness of the majority of women at childbearing age in the future.

However, this research does have some limitations. Above all, this study was only a descriptive analysis in order to investigate the spatiotemporal distribution of CHDs in Guangdong. Thus, it does not provide a definite causal relationship between the disease and those spatiotemporal features. In addition, because it is challenging to perform precise measurements of some exposures (e.g., occupational and environmental exposures), such potential factors were not included in this study, although they might affect the spatiotemporal distribution of the disease (4).

## Conclusions

In conclusion, our study analyzed a total of 8,653,206 perinatal infants to reveal the spatial-temporal and demographic characteristics of the prevalence of CHDs in Guangdong, China from 2016 to 2020. Further studies should be devoted to strengthening the prevention and treatment of CHDs, which will help reduce the prevalence of the disease and improve the life quality of patients.

## Data Availability Statement

The original contributions presented in the study are included in the article/Supplementary Material, further inquiries can be directed to the corresponding author/s.

## Ethics Statement

The studies involving human participants were reviewed and approved by Medical Ethics Committee of Guangdong Women and Children Hospital. Written informed consent from the participants' legal guardian/next of kin was not required to participate in this study in accordance with the national legislation and the institutional requirements.

## Author Contributions

YZ, HM, QZ, ZS, YX, LS, and DC: acquisition, analysis, or interpretation of data. HM, QZ, and ZS: drafting of the manuscript. HM: statistical analysis. DW and YZ: obtained funding and supervision. PG: anadministrative, technical, or material support. All authors: concept and design. All authors contributed to the article and approved the submitted version.

## Funding

The study was partly funded by the Project of the accounting of total health expenditure in Guangdong Province, the Philosophy and Social Sciences Research Project of Guangdong College (No. 2015WSYS0010), the Public Health Service System Construction Research Foundation of Guangzhou (2021–2023), and Medical Scientific Research Foundation of Guangdong of China (Nos. A2021094 and C2020032). The funder had no role in study design, data collection and analysis, decision to publish, or preparation of the manuscript.

## Conflict of Interest

The authors declare that the research was conducted in the absence of any commercial or financial relationships that could be construed as a potential conflict of interest.

## Publisher's Note

All claims expressed in this article are solely those of the authors and do not necessarily represent those of their affiliated organizations, or those of the publisher, the editors and the reviewers. Any product that may be evaluated in this article, or claim that may be made by its manufacturer, is not guaranteed or endorsed by the publisher.
