# Supplementary material for: Spatio-Temporal Distribution and Demographic Characteristics of Congenital Heart Defects in Guangdong, China, 2016–2020
Source: Front Public Health. 2022 Apr 26;10:813916. doi: 10.3389/fpubh.2022.813916 (PMC9086594; doi:10.3389/fpubh.2022.813916)

Supplementary Tables

Supplementary Table 1. Prevalence of total congenital heart defects in 21 cities of Guangdong from 2016 to 2020.

Region	City	Numbers of perinatal infants						Numbers of total congenital heart defects						Prevalence of total congenital heart defects (/10,000)					
		2016	2017	2018	2019	2020	Total	2016	2017	2018	2019	2020	Total	2016	2017	2018	2019	2020	Total
Guangdong	Total	1854646	1932464	1694639	1645752	1525705	8653206	10186	10479	10810	12032	10405	53912	54.92	54.23	63.79	73.11	68.2	62.3
PRD	Guangzhou	239118	283320	248077	247514	230031	1248060	2892	2598	2482	2042	2434	12448	120.94	91.7	100.05	82.5	105.81	99.74
PRD	Dongguan	140131	145055	128806	125161	104351	643504	161	589	536	710	609	2605	11.49	40.61	41.61	56.73	58.36	40.48
PRD	Shenzhen	232505	229823	213612	222875	278053	1176868	2063	1646	1368	2372	1701	9150	88.73	71.62	64.04	106.43	61.18	77.75
PRD	Huizhou	87364	89777	80782	79198	68600	405721	152	312	432	473	471	1840	17.4	34.75	53.48	59.72	68.66	45.35
PRD	Zhaoqing	55100	51414	40359	36668	30920	214461	110	89	149	148	99	595	19.96	17.31	36.92	40.36	32.02	27.74
PRD	Foshan	120334	139837	120635	114132	96340	591278	2244	2351	2974	3201	2241	13011	186.48	168.12	246.53	280.46	232.61	220.05
PRD	Zhongshan	62034	69290	61059	57773	48001	298157	1010	874	858	811	540	4093	162.81	126.14	140.52	140.38	112.5	137.28
PRD	Zhuhai	34600	37553	24789	29948	20865	147755	340	312	221	290	338	1501	98.27	83.08	89.15	96.83	161.99	101.59
PRD	Jiangmen	56239	67072	56684	49298	41950	271243	257	336	338	344	408	1683	45.7	50.1	59.63	69.78	97.26	62.05
Eastern Guangdong	Shanwei	50572	45481	43120	41456	37297	217926	33	13	12	8	11	77	6.53	2.86	2.78	1.93	2.95	3.53
Eastern Guangdong	Jieyang	90901	94674	92445	89321	71765	439106	73	203	200	160	48	684	8.03	21.44	21.63	17.91	6.69	15.58
Eastern Guangdong	Shantou	91387	86364	76879	75943	67112	397685	54	48	68	95	120	385	5.91	5.56	8.85	12.51	17.88	9.68
Eastern Guangdong	Chaozhou	38408	36847	29234	30226	25912	160627	7	6	13	10	18	54	1.82	1.63	4.45	3.31	6.95	3.36
Western Guangdong	Yangjiang	42140	42338	36049	33110	29010	182647	145	133	95	121	99	593	34.41	31.41	26.35	36.54	34.13	32.47
Western Guangdong	Maoming	116397	112382	99076	94287	88086	510228	138	159	245	324	341	1207	11.86	14.15	24.73	34.36	38.71	23.66
Western Guangdong	Zhanjiang	136685	137068	123429	118785	112885	628852	47	84	110	155	120	516	3.44	6.13	8.91	13.05	10.63	8.21
Northern Guangdong	Yunfu	41806	41338	35628	32424	29300	180496	34	63	52	42	50	241	8.13	15.24	14.6	12.95	17.06	13.35
Northern Guangdong	Qingyuan	62812	65758	56561	51384	45524	282039	123	194	328	300	327	1272	19.58	29.5	57.99	58.38	71.83	45.1
Northern Guangdong	Shaoguan	44374	46342	38964	34979	30291	194950	123	135	96	89	76	519	27.72	29.13	24.64	25.44	25.09	26.62
Northern Guangdong	Heyuan	48542	45096	38596	35648	31488	199370	113	180	122	220	262	897	23.28	39.91	31.61	61.71	83.21	44.99
Northern Guangdong	Meizhou	63197	65635	49855	45622	37924	262233	67	154	111	117	92	541	10.6	23.46	22.26	25.65	24.26	20.63

Supplementary Figures

Supplementary Figure 1. Prevalence of total congenital heart defects (CHDs) (per 10,000 perinatal infants) in Guangdong, China, 2016 - 2020.

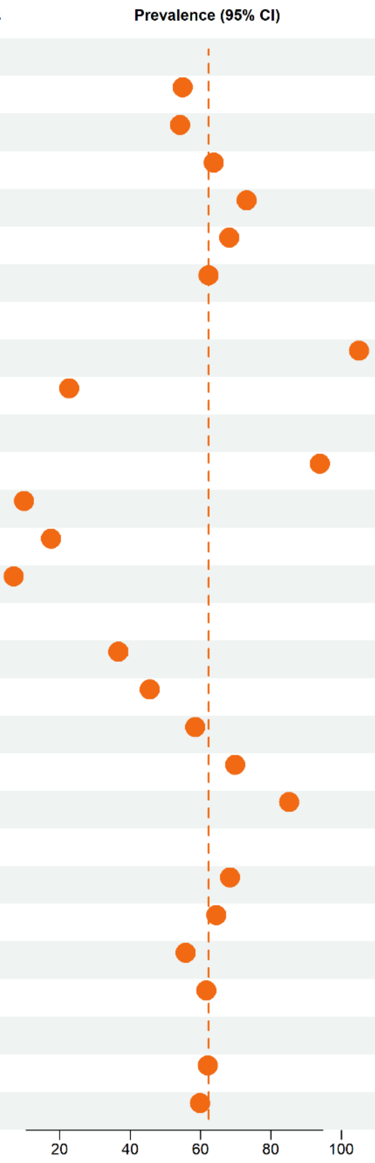

Subgroup	Numbers of perinatal infants	Numbers of total CHDs	Prevalence (95% CI)	P
Years				<0.001
2016	1854646	10186	54.92 (53.86~56.00)	
2017	1932464	10479	54.23 (53.20~55.27)	
2018	1694639	10810	63.79 (62.60~65.00)	
2019	1645752	12032	73.11 (71.81~74.42)	
2020	1525705	10405	68.20 (66.90~69.52)	
2016-2020	8653206	53912	62.30 (61.78~62.83)	
Residential areas				<0.001
Urban	4164278	43740	105.04 (104.06~106.02)	
Rural	4488928	10172	22.66 (22.22~23.10)	
Region of Guangdong				<0.001
PRD	4997047	46926	93.91 (93.06~94.76)	
Eastern GD	1215344	1200	9.87 (9.33~10.44)	
Western GD	1321727	2316	17.52 (16.82~18.25)	
Northern GD	1119088	3470	6.94 (6.72~7.18)	
Maternal age at delivery				<0.001
<20	236497	868	36.70 (34.32~39.21)	<0.001
20-24	1628600	7429	45.62 (44.59~46.66)	<0.001
25-29	3316539	19428	58.58 (57.76~59.41)	
30-34	2321374	16215	69.85 (68.78~70.93)	<0.001
≥35	1150196	9802	85.22 (83.55~86.92)	<0.001
Season of delivery				<0.001
Spring	1988879	13601	68.39 (67.24~69.54)	
Summer	2139333	13798	64.50 (63.43~65.60)	<0.001
Autumn	2387406	13318	55.78 (54.84~56.74)	<0.001
Winter	2137588	13195	61.73 (60.68~62.79)	<0.001
Infants' gender				<0.001
Male	4598644	28548	62.08 (61.36~62.80)	
Female	4053112	24288	59.92 (59.17~60.68)	

Supplementary Figure 2. Prevalence of total congenital heart defects (CHDs) (per 10,000 perinatal infants) of 21 cities of Guangdong, China in 2016 - 2020.

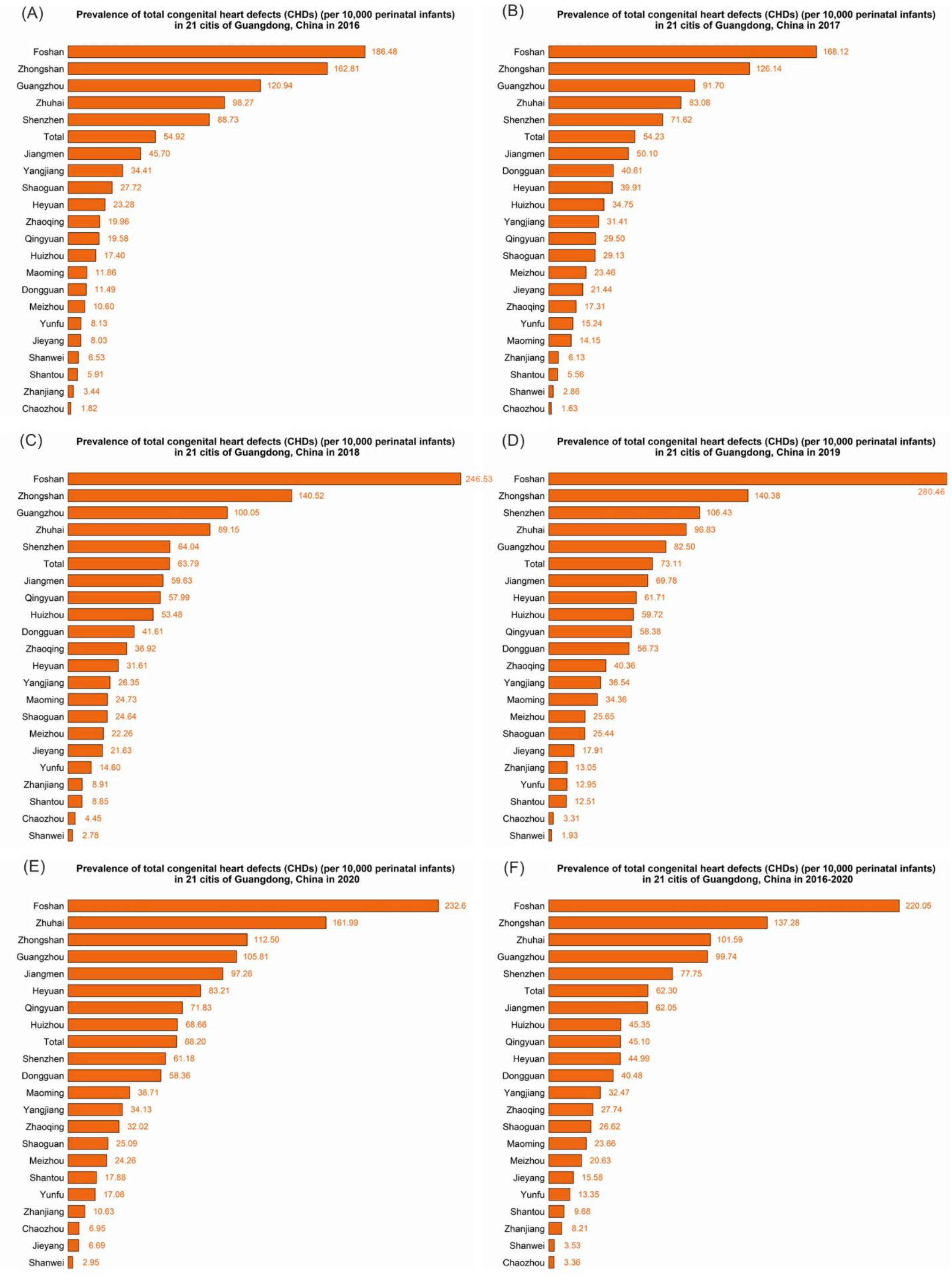

Supplement: Supplementary file 1 [file Data_Sheet_1.pdf]
